# COVID-19: challenges for dementia care and research

**DOI:** 10.1590/1980-57642020dn14-040002

**Published:** 2020-12

**Authors:** Marcia Cristina Nascimento Dourado, Tatiana Belfort, Alexandre Monteiro, Aline Tavares de Lucena, Isabel Barbeito Lacerda, Julia Gaigher, Maria Alice Tourinho Baptista, Michelle Brandt, Nathália Ramos Kimura, Natalie de Souza, Paula Gasparini, Rogéria Rangel, Valeska Marinho

**Affiliations:** 1 Center for Alzheimer's Disease, Institute of Psychiatry, Universidade Federal do Rio de Janeiro, Rio de Janeiro, RJ, Brazil.

**Keywords:** coronavirus infections, dementia, caregivers, behavioral symptoms, infecções por coronavírus; demência, cuidadores, sintomas comportamentais

## Abstract

The COVID-19 pandemic has raised significant concerns about the management and care for people with dementia and their caregivers. In this context, this work will discuss how social isolation or social distancing caused by the pandemic may impact the clinical management of people with dementia, caregivers’ health, and dementia research. The pandemic disrupts all forms of social interaction and may increase the behavioral impairment of people with dementia. Regarding pharmacological treatment, telemedicine is an option, but the context of social isolation raises questions about how to manage people with dementia with lack of cognitive stimulation and non-pharmacological treatment. In addition, the impact of the pandemic on caregivers should be considered. There is some evidence that telephone counseling can reduce depressive symptoms of caregivers of people with dementia. In dementia research, social isolation imposes researchers to modify their study protocols in order to continue collecting data by developing remote tools to assess the participants such as electronic informed consent and online questionnaires and tests. Thus, there is an urgent need for the evaluation and refinement of interventions to address several cognitive, behavioral, and clinical aspects of the long-term impact of the pandemic in dementia.

## RESUMO.

A pandemia causada pelo COVID-19 desencadeia grandes preocupações sobre o manejo e cuidados com as pessoas com demência e seus cuidadores. Neste contexto, discutiremos como o isolamento social causado pela pandemia pode impactar o manejo clínico de pessoas com demência, a saúde do cuidador e a pesquisa sobre demência. A pandemia interrompe todas as formas de interação social e pode causar aumento do comprometimento dos sintomas neuropsiquiátricos nas pessoas com demência. Em relação ao tratamento farmacológico, a telemedicina é uma opção, mas o contexto de isolamento social levanta questões sobre como manejar as pessoas com demência com falta de estimulação cognitiva ou intervenções nãofarmacológicas. Além disso, o impacto da pandemia sobre os cuidadores deve ser considerado. Existem evidências de que o aconselhamento telefônico pode reduzir os sintomas depressivos dos cuidadores. Além disso, o isolamento social impõe que pesquisadores modifiquem seus protocolos de pesquisa com o objetivo de continuar coletando dados, através do desenvolvimento de ferramentas remotas para avaliar os participantes, como o consentimento livre e esclarecido eletrônico e questionários e testes online. Assim, há uma necessidade urgente de avaliação e refinamento das intervenções para abordar aspectos cognitivos, comportamentais e clínicos do impacto de longo prazo da pandemia na demência.

## INTRODUCTION

Aged adults have been considered as the most vulnerable portion of the population at the onset of the coronavirus disease 2019 (COVID-19) pandemic. The COVID-19 pandemic has raised great concerns about the management and care for people with dementia and their caregivers.^1^ Furthermore, the effects of social isolation or social distancing may be detrimental to well-being, as they may increase people neuropsychiatric symptoms with dementia and caregivers’ burden, anxiety, and depression.^2^ For example, people with dementia may have difficulties in understanding the need for social isolation and procedures such as wearing masks or not having contact with their loved ones, aspects which may lead to changes in their behavior. In addition, the need for physical distancing results in increased isolation, lack of cognitive stimulation and physical exercise, decreased social engagement, and interruption of leisure activities.^3^ Thus, the pandemic highlights people with dementia vulnerability, due to an increase in their cognitive and behavioral impairment and a decrease in their social support.^3^

Social connectedness and social interaction are essential for health, well-being, and quality of life. An understanding and appreciation of the current and potential impact of the pandemic and the need for social isolation can help the development of strategies to assess the actual cognitive and behavioral functioning of people with dementia, as well as their caregivers’ health to improve their care. Therefore, in this context, we will discuss how social isolation or social distancing caused by the COVID-19 pandemic may impact the clinical management of people with dementia, the health of caregivers, and dementia research.

## CLINICAL MANAGEMENT OF PEOPLE WITH DEMENTIA

Dementia is characterized by multiple cognitive deficits, but one of the most challenging clinical manifestations of the disease is the management of behavioral and psychological or neuropsychiatric symptoms such as depression, wandering, personality changes, aggressiveness, delusions, accusations, hallucinations, and difficulty to sleep at night.^4^ ^,^ ^5^ Neuropsychiatric symptoms are frequent in dementia, affecting 80% of patients during the course of the disease.^4^ They have been described as having detrimental effects on the quality of life of both people with dementia and their caregivers.^4^ ^,^ ^5^

The pandemic caused by COVID-19 disrupts all forms of social interaction, possibly for a prolonged period of time, and it may increase people with dementia behavioral impairment. Most likely, social isolation affects people with dementia according to their disease severity: in mild individuals, one may observe an increase in their memory and orientation problems and a greater impairment of behavioral symptoms in moderate to severe dementia.

Social distancing under the COVID-19 pandemic has restricted access to community services for aged adults with dementia and their caregivers, which are largely shut down. A great concern is the access to these community services through different social and geographical contexts in countries such as Brazil. The nonattendance of face-to-face consultations or activities may worsen the cognition and overall functioning of this population, underling the need for health care providers and professionals to set up ways to ensure continuity of care^6^. Regarding the pharmacological treatment, telemedicine is an option, as it enables the clinician to provide care in the social distancing context.^2^ ^,^ ^3^ Evidence of the feasibility of telemedicine has been shown. A recent study compared the impact of additional services delivered to both care-recipient and caregiver through videoconference with telehealth targeted at caregivers by telephone-only during 4 weeks and found that telemedicine had averted the cognitive impairment evident in the telephone-only group.^6^ Varying degrees of improvement in physical and mental health, perceived burden, and self-efficacy were also observed among caregivers in the video-conferencing group.^6^ Nevertheless, some limitations and challenges of this kind of intervention should be addressed: lack of physical examination, reduced diagnosis confidence, lack of access to technology also related to social and geographical contexts, digital illiteracy, need for additional assistance to set up digital devices and connectivity problems and sensory impairment. Recognizing these limitations is crucial for the development of online alternatives to temporarily replace dementia-care programs.

Additionally, social isolation may hamper or interrupt access to well-known and effective non-pharmacologic treatments such as cognitive stimulation.^7^ Recently, a review highlights the current lack of high-quality evidence to determine whether assistive technology is effective in supporting people with dementia to manage their memory problems.^8^ The extent and duration of social distancing measures are uncertain and they may be significant during intermittent periods of time in some jurisdictions. Therefore, the context of social isolation raises questions about how to manage the lack of cognitive stimulation and non-pharmacological treatment for people with dementia neuropsychiatric symptoms. A combination of pharmacologic treatment and non-pharmacologic approaches is considered the gold standard for the appropriate management of the neuropsychiatric symptoms in dementia.^5^ Currently, there are informal reports on the effects of online cognitive stimulation, choral groups, and physical activities for people with dementia. Indeed, these new treatment technologies are a way to assess people with dementia in social isolation, but the development of formal programs with activities designed to alleviate the presence of neuropsychiatric symptoms is still necessary. Nevertheless, there are challenging questions to be answered in order to design effective non-pharmacological virtual programs: which activities are the most adequate ones? For how long and how to make people with dementia stay in front of a video, in a situation in which they may be confused and unable to understand and visualize the professional well. Most likely professionals will need to rely on caregivers to help them with the non-pharmacological virtual activities. How can professionals count on the help of caregivers, who may be in higher levels of distress? Also, studies on virtual reality-based intervention may help professionals and researchers to answer these questions, in order to mitigate social isolation impact on non-pharmacological interventions.^9^

## CAREGIVERS’ HEALTH

Most people with dementia depend on their caregivers for a living. Usually, caregivers of older adults with dementia report more stress, burden, and depression compared to caregivers of people with other diseases.^10^ Therefore, the impact of the pandemic and social isolation on formal and informal caregivers should be considered.

During social isolation, some families may face the need to care for people with dementia without help, as formal caregivers may be unavailable as they may become a vector of COVID-19 infection.^3^ In addition, social isolation interrupts access to daycare centers and community services for dementia. The daycare has been considered an important health technology in the configuration of support strategies for people with dementia and their caregivers.^11^ Proposed as a service model that provides a friendly and caring environment for people with dementia while caregivers take a break to decrease their exposure to primary stressors, the daycare center seeks to reduce the burden of care.^11^ ^,^ ^12^ Consequently, social isolation impacts on worsening the lack of a break from daily care in a context in which people with dementia may be presenting changes in their behavior.^3^

Studies on caregivers’ stressors have shown a connection between the context of care, the cognitive and behavioral status of the care recipient, the resources (formal and informal) used by the caregiver, and the transitions (or evolution) of the care situation.^13^ Thus, some caregivers may develop higher levels of anxiety and depression caused by the increased burden of care. This situation raises questions about how health professionals can support caregivers’ needs in the context of social isolation. Immediate actions should be taken in order to prevent the risk of domestic violence and abusive situations such as the development of digital programs and interventions aiming at the monitoring of domestic situations and the support of caregivers’ demands. There is some evidence that telephone counseling can reduce depressive symptoms and meets the important needs of caregivers of people with dementia.^14^ Also, a study suggests that online psychoeducational support and specific care guidelines can contribute to the well-being of individuals with dementia and caregivers.^15^ Therefore, digital interventions for caregivers’ anxiety, depression, and burden should include video and online psychoeducational programs, telephone calls, and messages to reach those with poorer digital resources.

Moreover, a better understanding of the buffering effects of social relationships during stressful events is necessary in the long run.^3^ The development of coping strategies in the social isolation context is fundamental for the management of caregivers’ psychological distress, in order to prevent dysfunctional coping strategies (confrontation, escape, and avoidance) that may have harmful consequences for the management of people with dementia.^16^

## DEMENTIA RESEARCH

Research activities are fundamental to understand the impact of the pandemic on people with dementia and their caregivers. Simulations suggest that social suppression will be needed for several months, with intermittent relaxing social distancing.^2^ ^,^ ^17^ Thus, there are many issues to be addressed regarding the feasibility to develop studies in this critical situation. For example, social cognition is the capacity to interpret and predict another's behavior according to beliefs, intentions, and emotions, and the ability to decode environmental stimuli to be better able to adapt to new situations.^18^ In dementia research, there are reports that people with Alzheimer's disease performed significantly worse than controls across all measures of social and emotional processing, such as recognition of social and emotional functioning.^19^ ^,^ ^20^ Certainly, people with social cognition dementia will be impacted by the increase of neuropsychiatric symptoms and the isolation context. Most likely, in a condition of lack of social interaction and cognitive stimulation, there will be a decrease in the social cognition of people with dementia, but how can clinical research measure this important variable and others such as cognitive functioning or neuropsychiatric symptoms in this context?

Currently, the most widely used methods of neuro-psychological assessment require hands-on manipulation of stimuli or carefully standardized administration of visual material.^21^ The social isolation caused by COVID-19 imposes researchers to modify their study protocols to continue collecting data by developing remote tools to assess the participants, such as electronic informed consent and online questionnaires and tests. For instance, Hantke and Gould^21^ reported the existence of an now online audiovisual version of the Montreal Cognitive Assessment (MoCA).

Measuring general cognition in social isolation is a key issue, but the development of tele-based measures of variables such as social cognition, neuropsychiatric symptoms, people with dementia's awareness of the situation, caregivers’ burden and quality of life are imperative to the advancement of research in this area. Besides, there will be challenges in terms of construct validity, adequate internet connection speeds, camera quality, privacy, measurement errors, and missing data that should be expected and need to be addressed and discussed in the context of the pandemic.^17^

The COVID-19 pandemic has led to a vigorous and multifaceted response from clinicians, health professionals, and researchers at multiple levels. The pandemic imposes the development of coping strategies and preventive interventions to support vulnerable groups such as people with dementia and their caregivers. Using digital technologies for caring and researching in dementia is a critical way to mitigate isolation, thus preventing morbidity in these at-risk individuals during the COVID-19 pandemic. Interventions that can be delivered under pandemic conditions to reduce the presence of people with dementia, neuropsychiatric symptoms and caregivers’ distress should be quickly boosted. There is an urgent need for the evaluation, and refinement of interventions to address several cognitive, behavioral, and clinical aspects of the long-term impact of the pandemic in dementia.

The COVID-19 pandemic has also significantly impacted current dementia research. The need for assessment, development of remote tools, and availability of resources for research should be improved in order to better understand the multiple impacts on people with dementia and in caregivers’ health and well-being as a way of supporting clinical practice and intervention development.
